# Educational strategies for preventing accidents in childhood: a systematic review

**DOI:** 10.1590/0102-311XEN036224

**Published:** 2024-10-18

**Authors:** Gabriela Garcia de Carvalho Laguna, Ana Luiza Ferreira Gusmão, Breno Oliveira Marques, Níkolas Brayan da Silva Bragas, Gabriela Alves de Souza Assis, Karolaine da Costa Evangelista, Natália Oliveira e Silva

**Affiliations:** 1 Instituto Multidisciplinar em Saúde, Universidade Federal da Bahia, Vitória da Conquista, Brasil.

**Keywords:** Accident Prevention, Child, Health Education, Health Promotion, Pediatrics, Prevenção de Acidentes, Criança, Educação em Saúde, Promoção da Saúde, Pediatria, Prevención de Accidentes, Niño, Educación en Salud, Promoción de la Salud, Pediatría

## Abstract

Accidents in childhood have a high morbidity and mortality rate and are often preventable, which reinforces the importance of educational measures to prevent unintentional injuries. This study aimed to identify and describe useful educational strategies for preventing childhood accidents in communities. This systematic review was guided by PRISMA (2020) and registered on the PROSPERO platform (ID: CRD42024500956). A search strategy was developed by combining the descriptors “Accident Prevention”, “Child”, and “Health Education” with the Boolean operator AND, applied to the PubMed/MEDLINE, Web of Science, LILACS, and SciELO databases. A total of 5,037 studies were located, including observational articles published from 2018 to 2023, with children aged 0-12 years and/or their parents/caregivers. The quality of the studies was assessed based on the *Qualitative Studies Checklist* and the *Research Triangle Institute Item Bank* instruments. The bibliographic sample consisted of 30 articles, mostly classified as high quality, with a population of 4,510 adults and 54,190 children from various countries. Educational strategies for accident prevention were described, aimed at parents and guardians, children, and both. This review, addressing innovative educational strategies for preventing childhood accidents, highlights playful approaches for children and visual methods for caregivers. Implementation faces challenges related to evaluation and socioeconomic factors, making rigorous criteria and prolonged follow-ups important for continuous effectiveness.

## Resumo

Acidentes na infância têm uma alta taxa de morbidade e mortalidade e, muitas vezes, podem ser evitados, o que reforça a importância de medidas educacionais para evitar lesões não intencionais. O objetivo foi identificar e descrever estratégias educacionais úteis para a prevenção de acidentes infantis no contexto da comunidade. Essa revisão sistemática foi orientada pelo PRISMA (2020) e registrada na plataforma PROSPERO (ID: CRD42024500956). Uma estratégia de busca foi desenvolvida combinando os descritores “*Accident Prevention*” (prevenção de acidentes), “*Child*” (criança) e “*Health Education*” (educação em saúde) com o operador booleano AND, aplicado às bases de dados PubMed/MEDLINE, Web of Science, LILACS e SciELO. Foram localizados 5.037 estudos, incluindo artigos observacionais publicados entre 2018-2023, com crianças de 0 a 12 anos e/ou seus pais/cuidadores. A qualidade dos estudos foi avaliada considerando os critérios dos instrumentos *Qualitative Studies Checklist* e *Research Triangle Institute Item Bank*. A amostra bibliográfica consistiu em 30 artigos, em sua maioria classificados como de alta qualidade, com uma população de 4.510 adultos e 54.190 crianças de vários países. Foram descritas estratégias educacionais para a prevenção de acidentes, voltadas para pais e responsáveis, crianças e ambos. Esta revisão, que aborda estratégias educacionais inovadoras para a prevenção de acidentes infantis, destaca abordagens lúdicas para crianças e métodos visuais para cuidadores. A implementação enfrenta desafios relacionados à avaliação e a fatores socioeconômicos, tornando critérios rigorosos e acompanhamentos prolongados importantes para a eficácia contínua.

## Resumen

Los accidentes en la infancia tienen una alta tasa de morbimortalidad y, muchas veces, son prevenibles, por lo que refuerza la importancia de medidas educativas para prevenir lesiones no intencionales. El objetivo de estudio fue identificar y describir estrategias educativas útiles para la prevención de accidentes en la infancia en el contexto comunitario. Esta revisión sistemática fue guiada por PRISMA (2020) y registrada en la plataforma PROSPERO (ID: CRD42024500956). Se realizó una búsqueda combinando los descriptores “*Accident Prevention*” (prevención de accidentes), “*Child*” (niño) y “*Health Education*” (educación en salud) con el operador booleano AND, aplicado a las bases de datos PubMed/MEDLINE, Web of Science, LILACS y SciELO. Se encontraron 5.037 estudios, que incluye artículos observacionales publicados entre 2018-2023, con niños de 0 a 12 años y/o sus padres/cuidadores. La calidad de los estudios se evaluó considerando los criterios de los instrumentos *Qualitative Studies Checklist* y *Research Triangle Institute Item Bank*. La muestra bibliográfica consistió en 30 artículos, en su mayoría clasificados como de alta calidad, realizados con una población de 4.510 adultos y 54.190 niños de varios países. Se describieron estrategias educativas para la prevención de accidentes dirigidas a padres y tutores, niños o ambos. Esta revisión aborda estrategias educativas innovadoras para la prevención de accidentes en la infancia y destaca los enfoques lúdicos para los niños y las técnicas visuales para los cuidadores. La aplicación se enfrenta a desafíos relacionados con la evaluación y los factores socioeconómicos, por lo que los criterios estrictos y los seguimientos prolongados son importantes para mantener su eficacia.

## Introduction

Accidents involving children are mostly unintentional and have high rates of morbidity and mortality, making them one of the leading causes of death among 5-14-year-olds. They can also have serious consequences, potential long-term impacts, and affect the individual’s quality of life. These unintentional injuries can result from traffic accidents, falls, drowning, burns, poisoning, asphyxiation, or sports practices. They affect children from all living conditions but are less frequent among families with higher economic status and levels of education. Developing countries show greater economic burden and mortality statistics due to these accidents ^1^ ^,^ ^2^ ^,^ ^3^.

Investing in prevention and improving these efforts is crucial to minimize preventable injuries. Multimodal educational interventions should prioritize children’s knowledge, attitudes, and safer behaviors ^1^ ^,^ ^2^ ^,^ ^3^. These interventions can take the form of classroom teaching, family counseling, lectures, and educational materials such as posters, messages, stories, videos, games, virtual reality approaches, and more. However, there is a need for systematization and evaluations of the impact of these strategies, as the literature currently lacks such assessments ^3^ ^,^ ^4^.

Considering the impact of accidents in childhood, some systematic reviews have already been developed and have contributed to understanding injuries, quality of life impacts, environmental change strategies to prevent them in general, and some cause-specific prevention ^2^ ^,^ ^3^ ^,^ ^5^ ^,^ ^6^. This research aims to contribute to the theme by identifying and systematically describing useful educational strategies for preventing childhood accidents in the community context.

## Methodology

This systematic review was guided by the research question: “What educational strategies (I) can be employed to prevent accidents in a community context (Co) during childhood (P)?”. The question was formulated according to the PICo strategy (Population, Interest/phenomenon of interest, and Context) ^7^, due to its qualitative approach. For its development, the criteria of the PRISMA (*Preferred Reporting Items for Systematic Reviews and Meta-Analyses*) ^8^ were followed, and a protocol for this review was registered on the PROSPERO platform (protocol n. CRD42024501952).

We searched for articles published from 2018 to 2023 that focused on educational strategies and intervention programs for accident prevention in community settings. We included studies involving children aged 0-12 years and/or their parents/caregivers, while excluding studies that did not meet the inclusion criteria, were incomplete, or did not address the research question. We excluded studies centered on patient safety in hospital settings rather than community settings and literature reviews. Language was not a criterion for exclusion. We accessed articles that were not fully available whenever possible, either via the institution of the authors of this research or directly from the authors of the studies.

The following descriptors were combined into a search strategy using the Boolean operator AND, and applied to the Web of Science, PubMed/MEDLINE, LILACS, and SciELO databases in January 2024: “Accident Prevention”, “Child”, and “Health Education”. For screening studies, initially through titles and abstracts, followed by full-text reading of eligible studies, the free web platform Rayyan (https://www.rayyan.ai/) was used. This stage was conducted by two independent and blinded evaluators (G.G.C.L. and A.L.F.G.), with disagreements resolved by consensus. The selected studies were managed for data extraction and analysis using Microsoft Excel (https://products.office.com/). The extracted data included author and year of publication, study design and location, sample size, key findings, and limitations.

The qualitative studies were evaluated using the *Qualitative Studies Checklist* ^9^, an instrument proposed by the Critical Appraisal Skills Programme (CASP). Articles were classified into the following categories: (i) high methodological rigor, for meeting at least nine out of 10 items on the checklist and (ii) moderate methodological rigor, for meeting five to eight items. Regarding quantitative studies, six criteria from the *Research Triangle Institute Item Bank* (RTI-Item Bank) ^10^ were evaluated: (i) clearly defined inclusion and exclusion criteria; (ii) use of valid and reliable measures to assess inclusion and exclusion criteria; (iii) standardized recruitment strategy for study participants across all groups; (iv) appropriate sample selection; (v) results assessed using valid and reliable measures consistently implemented for all study participants; (vi) consideration of confounding and effect-modifying variables in the study design and/or data analysis. Articles were classified as: (i) high methodological rigor, for meeting five to six items of the criteria or (ii) moderate methodological rigor, for meeting three to four of them.

The quantitative information from the research was presented using descriptive statistics, including absolute numbers and percentages. Qualitative data were summarized in individual study synthesis figures.

## Results

**Figure 1:**
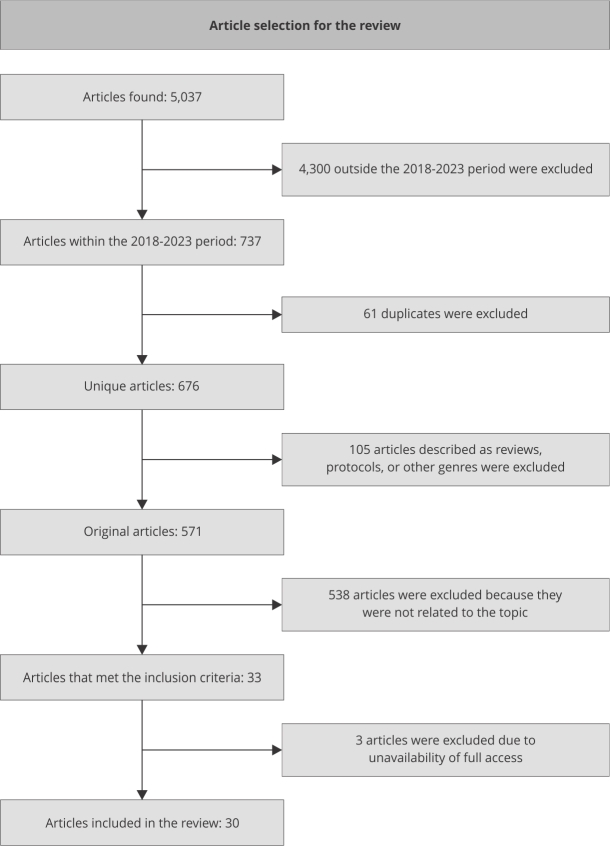
Study selection flowchart.

Source: prepared by the authors.

A total of 5,037 records were found in the searched databases. Out of these, 737 were published from 2018 to 2023 and were screened based on eligibility criteria. After applying the criteria, 30 articles were selected for analysis, as illustrated in Figure 1.

The following countries were represented among the studies: the United States (30%), Brazil (9.99%), Iran (6.66%), Saudi Arabia (6.66%), China (3.33%), South Korea (3.33%), Egypt (3.33%), Spain (6.66%), Australia (3.33%), Bangladesh (3.33%), Israel (3.33%), Pakistan (3.33%), the United Kingdom (3.33%), Thailand (6.66%), Turkey (3.33%), and Vietnam (3.33%). The studies consisted of 18 qualitative and 12 quantitative research. The study population included approximately 4,510 adults - parents and caregivers of children under 12 years old - and 53,987 children, aged from 3 months to 12 years old. Interventions for the prevention of various topics were included, with 11 studies focusing on accidents and unintentional injuries (33.66%), six on interventions for traffic safety (20%), five on home risk situations (16.66%), five on fall prevention (16.66%), five on correct car seat usage (16.66%), three on drowning prevention interventions (9.99%), three on fire prevention (9.99%), one on poisoning and intoxication prevention (3.33%), one on firearm accidents (3.33%), and one on injury identification (3.33%). It is important to note that some studies addressed more than one topic. Box 1 shows the study characterization.

**Box 1:** Individual study characterization: author, year, design, study location, sample, main results, and quality analysis outcome.

STUDY (YEAR)	DESIGN (STUDY LOCATION)	SAMPLE	MAIN RESULTS	QUALITY
Ahmad et al. ^28^ (2018)	Intervention study (Pakistan)	207 children aged 8 to 16 years of both genders	A children’s storybook was used to initiate a discussion with primary school children about preventing traffic accidents. A baseline test was conducted before the discussion, and 2 tests were conducted immediately after the discussion and 2 months later. The results showed a statistically significant improvement in mean scores over time based on gender, grade, and school type (p-value < 0.001)	A
DelCastillo-Andrés et al. ^29^ (2018)	Exploratory and quasi-experimental study (Spain)	122 children aged 10 to 12 years of both genders	The Safe Fall program is designed to lower the risk of unintentional falls in school-age children. The program was assessed before and after implementation, and the results indicate an improvement in the correct positioning of protective measures across all variables. The difference between the pre- and post-intervention was statistically significant (p < 0.05) for all 5 responses	A
El Selfi et al. ^22^ (2018)	Intervention study (Egypt)	244 women aged 19 to 38 years with at least one child aged 1 to 5 years	A health education intervention addressed child safety and injury prevention using PowerPoint presentations, explanatory videos, models, pamphlets, and discussions. The pre- and post-intervention questionnaires showed changes in knowledge (34.8%), attitude (18.5%), and self-efficacy (31.1%). There was a significant increase in the average post-intervention score (p < 0.001), except for the attitude towards immediate first aid measures (p = 0.078)	B
Kahriman & Karadeniz ^23^ (2018)	Quasi-experimental study (Turkey)	300 women with an average age of 31 years with children aged 0 to 6 years	Mothers were taught to be more aware of safety precautions related to various types of pediatric injuries. Two tools were used for this purpose: the *Safety Precautions Identification Scale for Prevention of Pediatric Injuries by Mothers of Children Aged 0 to 6* and the *Pediatric Injury Risk Assessment Form* (RAF). The average scores on both tools showed a significant increase in the pediatric injury identification scale (p = 0.000) and RAF (p < 0.05). Furthermore, there was a significant difference in scores on the pediatric injury identification scale based on the mother’s education level, the child’s age, the number of children, and the mother’s employment status	A
Kuroiwa et al. ^16^ (2018)	Single-blind randomized clinical trial (United States)	213 participants aged 30 to 35 years of both genders	A study was conducted to compare the effectiveness of traditional lecture-style classes on child passenger safety with rapid and simple social learning using theoretical and practical explanatory videos. The study found that the percentage of correct car seat installations increased from 17% to 52% in the traditional lecture-style classes and from 16% to 50% in the social learning group	A
Machin et al. ^19^ (2018)	Qualitative action research (Thailand)	8 couples (8 men and 8 women), aged 24 to 54 years, with children up to 4 years old	Analysis of parents’ perceptions of a collaborative program aimed at preventing accidental home injuries in children and its influence on their behavior showed that the intervention improved their children’s injury-prevention behavior at home	A
Schwebel et al. ^31^ (2018)	Nonrandomized trial (China)	56 children aged 8 to 10 years of both genders	A study was conducted to improve pedestrian safety using a smartphone-based virtual reality program in a simulated environment. The study found that the probability of accidents decreased from 0.40 to 0.09 post-training. Observational data also showed an increase in the chances of pedestrians looking at traffic in the opposite direction when crossing the first traffic lane (OR = 2.4)	B
Silva et al. ^24^ (2018)	Descriptive and intervention study (Brazil)	20 mothers aged 16 to 25 years	Games with illustrations related to childcare themes were created. The first post-intervention testing showed a significant improvement in knowledge scores. However, in the second test (5 months after the intervention), the scores decreased compared to the first post-test, particularly about the applied thematic criteria	A
Ramos et al. ^32^ (2018)	Intervention study (Vietnam)	40,198 questionnaires from children aged 5 to 11 years of both genders	An educational session was held in schools to address the dangers of aquatic environments. The session included practical training on how to float in water and essential water safety skills. Interactive games were conducted, and prizes were distributed to the winners. The intervention was found to have a significant positive impact, as observed through a questionnaire application (p < 0.001)	A
Wang et al. ^20^ (2018)	Intervention randomized trial (United States)	277 women with an average age of 27.3 years	During the study, different strategies were discussed for preventing fire, poisoning, and falls based on the Social Cognitive Theory, which focuses on community support. After a 6-month follow-up, the intervention group was able to reduce the number of safety issues, and this reduction was sustained at the 12-month mark (p = 0.035)	A
White et al. ^37^ (2018)	Randomized clinical trial (Australia)	611 children aged 5 to 7 years and 357 parents or caregivers aged 25 to 66 years of both genders	The Learn to Be Safe with Emmy and Friends program was compared to a waitlist condition. The program was effective in improving children’s knowledge and skills related to interpersonal safety, as evaluated by their parents. These benefits were sustained even after 6 months of the program, with participating children expressing greater confidence in disclosing sensitive information. However, the program had no significant impact on children’s safety identification skills, disclosure intentions, or their ability to interact safely with others	A
DelCastillo-Andrés et al. ^30^ (2019)	Randomized clinical trial (Spain)	439 children aged 6 to 12 years of both genders	The Safe Fall program was carried out as part of physical education classes, and the performance of balance exercises was compared. The risk of injury was evaluated before and after the program implementation. It was observed that over 90% of students were at a high risk of injury because of their natural response to falls. However, after the program, the risk percentage reduced significantly, ranging from 8.7% to 18.3%	A
Freitas et al. ^34^ (2019)	Experimental study with descriptive and analytical approach (Brazil)	173 children (average age: 9.8 years old) of both genders	The educational strategy was designed to encourage children to ask questions and share their ideas about traffic perception. This was done by using multiple suggestions raised by children’s drawings. The results showed a significant improvement in the experimental group’s knowledge (p < 0.05) compared to the control group. However, no significant difference in practices and attitudes between the 2 groups was identified	A
Macy et al. ^11^ (2019)	Randomized pilot test (United States)	339 parents of both genders aged 18 to 29 years	In this study, 4 different interventions were compared to see which was most effective. These interventions were: (1) a generic information sheet; (2) a personalized brochure sent by mail after the ER visit; (3) a single session of motivational interview-based counseling in the ER; and (4) a complete intervention (counseling and brochure). The results showed that parents who received the complete intervention (counseling and brochure) had an increase (+6.12%) in the proportion of children using an appropriately sized car seat at the 6-month follow-up. On the other hand, the other groups showed a decrease (-1.69 to -9.3%) in the proportion of children using the appropriate car seat	B
McLaughlin et al. ^35^ (2019)	Mixed-methods study (California, United States)	1,424 and 1,522 children for pre- and post-test and 250 and 369 children for pre- and post-intervention crossing behavior analysis, aged 5 to 11 years, both genders	An educational program was implemented by immersive experiences to encourage safe behavior while crossing the street. Results showed a significant increase in correct responses for 9 out of the 10 questions (p < 0.01). Furthermore, there was a marked improvement in the number of children who looked both ways before crossing the street (41% vs. 10%, p < 0.001)	A
Nascimento et al. ^25^ (2019)	Interventional research with primary data (Brazil)	30 caregivers of both genders with children	A figure was used as a trigger for dialogue exposure about accident risk situations. Before the action, 187 risk situations were identified. After the action, the number increased to 215. Additionally, the mean of discriminated situations increased to 8.7 (SD = 2.60). The Wilcoxon Test showed a statistically significant change (p < 0.001)	A
Foulds et al. ^38^ (2021)	Quantitative study (Bangladesh)	1,177 children and 776 caregivers	The mentoring model of Play Safe with Sisimpur program was created to enhance injury prevention knowledge and encourage behavior change. According to research findings, there was a significant increase in the knowledge related to injury prevention and treatment of various types of injuries such as burns, electrocution, falls, home risks, safe play, traffic injuries, and water safety. Additionally, the research results indicate that adults also demonstrated an increase in their injury prevention knowledge, specifically related to animal bites, burns, falls, home risks, and traffic injuries	B
Wang et al. ^26^ (2020)	Intervention randomized trial (United States)	277 biological pairs of babies and mothers, mothers over 18 years old, and babies aged 12 to 32 months	The intervention was designed to prevent fire and falls, control poisoning, and promote the use of car seats. It was conducted by group sessions that aimed to help families develop skills and new perceptions about child safety. The intervention also provided social support and helped build self-efficacy. The study found that the intervention was more effective in families with multiple home safety problems than in those with few or no problems. The effect size was larger in the former group (Cohen’s d = 0.99) than in the latter group (Cohen’s d = 0.15)	A
Budziszewski et al. ^17^ (2021)	Analytical and intervention study (United States)	200 caregivers of both genders	The Child Car Seat Program intervention was developed to increase caregivers’ knowledge about the use and importance of car seats. Before the course, caregivers correctly answered an average of 46% of the questions (M = 3.25, SD = 1.46). Notably, the average correct responses increased to 73% (p < 0.001) after the course	B
Choi & Ahn ^12^ (2021)	Controlled and randomized clinical trial (South Korea)	167 participants aged 30 to 39 years of both genders	The study aimed to compare the effectiveness of 2 types of intervention and control regarding unintentional childhood accident prevention. The first intervention was a virtual program containing interactive quizzes, while the second intervention was the same program content in PDF format. After the intervention, there was no significant difference in safety knowledge between the groups. However, when it came to safety practices, there was a statistically significant difference, with the software group showing the highest improvement, followed by the document group, and the control group showing the least improvement. The mean values were 3.52 ± 0.28, 3.51 ± 0.28, and 3.32 ± 0.25, respectively (p < 0.001)	A
Furman et al. ^18^ (2021)	Prospective observational study (United States)	50 parents and caregivers over 18 years old of both genders	The impact of the Mobile Safety Center was evaluated by 1 pre- and 3 post- intervention tests (immediate, after 4 weeks, and after 6 months of intervention). During the event, a trained educator presented various safety situations in different areas of the home. After the presentation, participants were tested to evaluate their preparedness for emergencies. The results showed that participants were more likely to have a fire escape plan after the presentation (post-test 1) than before (pre-test), with a significant p-value (0.014). Additionally, participants were more likely to have the poison control number readily accessible after post-test 1 compared to pre-test (p = 0.002) and post-test 2 compared to pre-test (p < 0.001)	B
Gesser-Edelsburg et al. ^39^ (2021)	Multimodal qualitative study (Israel)	101 children aged 3 to 10 years, and 303 adults with an average age of 34 years of both genders	A hybrid intervention model was implemented to decrease the incidence of unintentional injuries in the Bedouin community. The model involved three approaches: Positive Deviance (PD), Community-Based Participatory Research (CBPR), and Entertainment Education (EE) based on Bedouin theatrical traditions. The model led to the emergence of various PD ideas and practices to prevent and avoid childhood injuries. Additionally, a safe children’s playroom was created in a neighborhood mosque, as well as cross-learning and cascading learning networks were established among Bedouin community members spread across multiple locations	B
Moridi et al. ^13^ (2021)	Quasi-experimental study (Iran)	200 women with an average age of 30 years with children under 5 years old	An educational program was conducted via WhatsApp group sessions in the form of lectures, Q&A, group discussions, and the use of educational images, video clips, and PowerPoint. The program was based on the health belief model regarding accident prevention behaviors. Following the program, the group’s knowledge and behavior improved in almost all areas, except for perceived barriers	A
Kusol et al. ^36^ (2022)	Quasi-experimental study (Thailand)	120 children aged 7 to 12 years of both genders	The Potential Support Program on Drowning Prevention is based on the concept of social support. It emphasizes the promotion of students’ potential by working with their teachers to develop positive knowledge, skills, and attitudes toward self-prevention against drowning. An assessment form was used as a pre-and post-test to evaluate the program effectiveness. Comparing the mean differences in drowning prevention potential between the experimental and control groups after participation in the program, the experimental group showed a statistically significant increase (p < 0.001)	A
Temsah et al. ^27^ (2022)	Experimental study (Saudi Arabia)	308 adult participants of both genders	A pre- and post-test questionnaire was applied, and the intervention was a childhood and adolescence safety knowledge campaign. The knowledge score improved from 36.2 (SD = 17.7) to 79.3 (SD = 15.6) after the campaign participation (p < 0.001). Both the perception of accident avoidability (p < 0.001) and the usefulness of educational campaigns by parents improved (p < 0.001)	A
Bakhurji et al. ^14^ (2023)	Intervention study (Saudi Arabia)	303 adult participants of both genders	An online module was tested to increase caregivers’ awareness and knowledge about car seats. The module included educational videos, images, and recommendations. The average pre-test knowledge score of 11.64 significantly increased to 13.1 in the post-test (p < 0.001)	A
Estebsari et al. ^21^ (2023)	Quasi-experimental study (Iran)	70 women with children up to 5 years old	An educational program aimed at reducing the risk of home accidents, based on the *Home Accident Prevention Guide* from the Iranian Ministry of Health, was evaluated using pre- and post-test questionnaires. Before the intervention, there was no significant difference between the 2 groups (p > 0.05). However, after the intervention, there was a significant difference between the intervention and control groups	A
Kendi et al. ^15^ (2023)	Randomized controlled pilot study (United States)	60 participants aged 30 to 34 years of both genders	During the study, additional virtual demonstrations of infant car seats were provided at 3 and 6 months of age, in addition to the traditional in-person verification only at 9 months. In each session, the family installed the seat and child position, and some safety technical parameters were assessed together. Errors were noted and corrected. At the end of the study, the confidence and acceptability measures were similar between groups. However, the intervention group achieved a greater reduction in error rate compared to the control group, although the error rate was reduced in both groups (control group: 7% ± 4; intervention group: 2% ± 3; p < 0.001)	A
Taylor et al. ^40^ (2023)	Intervention study (United Kingdom)	762 families with children aged 3 to 7 months of both genders	The study involved implementing evidence-based home safety promotion strategies, such as safety messages, activity sessions, and home safety checklists. The primary goal was to ensure that families had a working smoke alarm, stored poisons out of reach, and had a stairgate in place. After 24 months, there was no significant difference between groups regarding primary outcome (55.8% vs. 48.8%) or rates of medically attended injuries. However, families who received the intervention were more likely to store poisons safely (OR = 1.81, 95%CI: 1.06-3.07), have a fire escape plan (OR = 1.81, 95%CI: 1.06-3.08), use a fireguard, or have no fire (OR = 3.17, 95%CI: 1.63-6.16), and perform more safety practices	A
Teichman et al. ^33^ (2023)	Retrospective cross-sectional study (United States)	8,832 children aged 6 to 8 years of both genders	The Safety Ambassador program employs creative and interactive teaching methods to educate children in areas of injury risk, such as traffic and car safety, wheeled sports and helmets, and fall prevention. As a result of these classes, 1st-grade students demonstrated an improvement in their average knowledge score from 9 in the pre-test to 9.8 in the post-test (p < 0.01). Behavior modification scores increased significantly from an average pre-test score of 3.2 to 3.6 in the post-tests (p < 0.01). Additionally, 2nd-grade students improved in both knowledge (p < 0.01) and behavior (p < 0.01)	B

95%CI: 95% confidence interval; ER: emergency room; OR: odds ratio; SD: standard deviation.

Source: prepared by the authors.

### Interventions aimed at caregivers

Most interventions were conducted by professionals with a background in Public Health and Pediatrics in a community setting, with only one conducted in a hospital context, despite the educational project focusing on the community setting ^11^.

Some interventions used digital media through software, virtual sessions, and electronic content ^12^ ^,^ ^13^ ^,^ ^14^ ^,^ ^15^. Creating virtual programs for preventable accidents with interactive quizzes led to increased safety practices. Sessions with videos, images, recommendations, and practical virtual demonstrations increased knowledge and awareness of the importance of traffic safety, ultimately enhancing the correct use of child seats regarding installation and positioning ^12^ ^,^ ^14^ ^,^ ^15^. Conference-style presentations with practical sessions were also employed to address this topic ^16^ ^,^ ^17^. Content delivery primarily involved lectures directed at parents and caregivers of children ^16^ ^,^ ^17^ ^,^ ^18^.

Behavioral approaches based on Social Cognitive Theory enabled the reorganization of habits, diminishing home safety issues and extending knowledge beyond the family context to encompass the community as a whole ^19^ ^,^ ^20^. An intervention based on the Health Belief Model, addressing participants’ perceptions of health-related risks, benefits of habit change, and barriers to habit change, resulted in reduced perceptible barriers and increased awareness, sensitivity, severity, and benefits related to habit change, measured using accident prevention questionnaires ^13^ ^,^ ^21^.

The combination of intervention strategies such as presentations, group messaging apps, videos, brochures, practical sessions, and counseling sessions showed positive outcomes for increasing knowledge and safety practices in childhood, measured using pre- and post-test questionnaires, and led to a significant difference in identifying pediatric injuries and their risks ^11^ ^,^ ^13^ ^,^ ^22^ ^,^ ^23^.

The less frequent playful interventions for children’s caregivers, such as illustrated games related to childcare themes, improved knowledge indices immediately after the first intervention immediately after the intervention. However, in the second test, structured similarly to the first, applied five months post-intervention, there was a reduction in the number of mothers with optimal scores on the childhood accident prevention questionnaire ^24^.

Group sessions, using triggering figures for discussion and meetings for skill development, as well as encouragement regarding children’s safety situations, increased the identification of childhood accident risk situations. Group dynamics on fire prevention, poison control, and car seat use facilitated community support and self-efficacy, being more effective for families with various home safety issues ^25^ ^,^ ^26^.

Educational and governmental accident risk training programs produced knowledge about childhood and adolescent safety, addressed by a significant increase in post-intervention perception of accident preventability. Additionally, caregivers reported the perceived effectiveness of campaigns in improving child safety ^21^ ^,^ ^27^.

### Interventions aimed at children

All interventions for this audience were implemented within schools, in some cases including both public and private institutions, by researchers with interests in health management ^28^, educational assessment ^29^ ^,^ ^30^, applied health psychology ^31^, prevention ^32^ ^,^ ^33^, and pediatric health education ^34^ ^,^ ^35^ ^,^ ^36^.

Activities with practical components yielded positive results in increasing children’s knowledge about accident prevention. Simulating an urban traffic environment, for example, whether using virtual reality or immersive life-sized settings, showed that practical experiences in traffic were effective in increasing the likelihood that children would adopt responsible and preventive behaviors when crossing the street, as well as in identifying risk patterns and reducing the chance of accidents. Theoretical-practical educational sessions addressing the dangers of aquatic environments were able to increase knowledge about risk attitudes and water safety skills ^31^ ^,^ ^32^ ^,^ ^35^. In this regard, recognized programs such as Safe Fall also brought significant changes, reducing the risk of injury and increasing the adoption of safe positions in the event of a fall ^29^ ^,^ ^30^.

Interactive games, educational and playful resources, and the use of active methodologies were also crucial in acquiring new knowledge on unintentional injury prevention. Programs like the Safety Ambassador and the Drowning Prevention support program used creative approaches, educational materials, and manuals to effectively enhance student knowledge ^28^ ^,^ ^33^ ^,^ ^34^. Similarly, playful tools were used by a program that aimed to improve students’ knowledge, skills, and positive attitudes toward preventing aquatic accidents ^36^.

### Interventions for mixed audiences

The interventions applied to both adults and children were predominantly conducted by professionals interested in health education and behavioral science ^37^ ^,^ ^38^ ^,^ ^39^ ^,^ ^40^, primarily within a school setting, but with one instance in a healthcare and social assistance service ^40^ and another involving focus groups from the studied community ^39^.

Television programs were designed/adapted to enhance knowledge among adults and children regarding injury prevention and risk behavior change, proving widely effective in improving understanding among children and adults on preventing and treating burns, electrocution, falls, household hazards, safe play, traffic injuries, as well as enhancing knowledge and skills in interpersonal safety ^37^ ^,^ ^38^.

Other actions were designed for implementation within the family context. Promotion of evidence-based home safety was implemented using various methods that addressed educational topics aimed at reducing the risk of common causes of injuries ^40^. Although the intended primary outcome and rates of medically attended injuries were not affected by the intervention, families were more inclined towards safety behaviors and practices. Cultural differences were also addressed using hybrid model interventions for unintentional injury reduction ^39^. By incorporating traditional theatrical elements of the target ethnic group, the tools were able to generate ideas and positive diversion practices for injury prevention, as well as creating safe play spaces for children.

The quality assessment of the studies was conducted using the *Qualitative Studies Checklist* for qualitative studies and the RTI-Item Bank for quantitative studies. Among the studies, 22 (73.33%) were classified as high quality, and eight (26.66%) as moderate quality.

## Discussion

This research highlights the growing demand for playful and interactive educational strategies aimed at preventing childhood accidents, which can be targeted at both caregivers or children, with varied approaches according to the context, skills, and specific needs of each target audience. Therefore, more playful and engaging approaches directed at children are described, such as stories and games designed to teach and reinforce learning, considering the specific cognitive abilities of each age group in the childhood cycle. Whereas, actions formulated for caregivers aimed to solidify more technical knowledge, predominantly visually, with group interactions, time-efficient activities, and methodologies involving lectures, quizzes, and administered questionnaires. The results also demonstrate difficulties in implementing these programs due to multiple factors related to the persistence of high rates of childhood accidents, such as the difficulty of maintaining and evaluating these actions in the long term, as well as issues related to parents’ level of education, socioeconomic vulnerabilities, and the geographical or cultural context in which some studies were conducted.

Educational technologies enable access to different intelligences and skills, incorporating both written and non-written language, resulting in greater engagement of users and professionals with the topics ^41^. Thus, the process of childhood teaching and learning allows for the educational development of children in orientation, structuring, motivation, problem-solving, and creativity perspectives. Therefore, playfulness in early childhood education allows for different possibilities of expression, analysis, and critical thinking of students, facilitating the understanding of adult reality from the child’s perspective and thereby stimulating autonomy and decision-making ability ^42^. From this understanding, art is reaffirmed as an important tool for learning and for the development of long-term safer habits. Thus, it mobilizes affective and emotional aspects alongside theoretical knowledge to promote redefinitions and new interactions capable of fostering the prevention of various issues ^43^.

The importance of storytelling during this period involves establishing an emotional bond between the story and the elements specific to the contexts experienced by children, being a psycho-pedagogical resource that amuses contrast to the monotony of the traditional teaching model ^44^. The use of a children’s storybook sparked a discussion among elementary school children about traffic accident prevention. It acted effectively toward this goal, as there was a statistically significant improvement in the average scores obtained by the children in tests administered immediately after the discussions and another conducted two months later. The latter showed a reduction in obtained values compared to the first post-intervention test, corroborating the need for continuous education to maintain the learning of safety measures ^28^. The article, featuring innovative modeling due to the didactic strategy applied by a pictorial storybook, employs a methodology based on a learning assumption during a primary school academic year to calculate the sample size, followed by thematic discussions grounded in this expected knowledge, which may be recognized as a factor of ambiguity. The use of an unvalidated questionnaire tends to elicit initial impressions that warrant further analysis for recognition. Furthermore, schools showed a statistically significant increase in average scores over time (p-value < 0.001) ^13^.

Prevention strategies based on traditional theoretical or theoretical-practical teaching methods, by cycles of classes, lectures, and workshops also proved effective in generating new knowledge and skills to recognize and deal with potentially dangerous situations for children. Knowledge of the location of possible accidents, circumstances, and mechanisms of injuries essential to prevent such events from occurring. Thus, changes in focus on dangers in domestic environments, for example, tend to reduce risks of falls, poisonings, and burns by taking precautions to make it difficult for infants to access toxic or harmful materials ^45^. From this perspective, providing guidance to caregivers, making adaptations to residential spaces, and identifying risk factors, especially considering the child’s developmental stage and common behavioral habits for the age group, are crucial to developing effective interventions ^46^.

Various actions combine traditional teaching methodology with technological tools, such as explanatory videos, group discussions, video clips, apps, and quizzes, yielding positive outcomes in some studies, given the established statistical significance and feedback received ^13^ ^,^ ^17^ ^,^ ^19^ ^,^ ^22^ ^,^ ^23^. On the other hand, other studies demonstrate that the use of alternative techniques - employing novel tools - shows similar effectiveness to the traditional format. Short video lessons presenting car seats specifications and steps for their installation seem to be similarly well understood in the short digital teaching format. It tends to increase participant’s adherence and practicality for individuals can watch them on demand, investing less time and therefore making it easier to implement the acquired teachings later on ^14^ ^,^ ^16^.

The integration of technology into intervention programs has yielded promising results, particularly in vehicle traffic safety. The strategy involving virtual demonstrations with certified child passenger safety technicians (CPSTs) and families for checking car seats resulted in fewer installation errors compared to the control group, which only participated in in-person demonstrations (control group: 7% ± 4; intervention group: 2% ± 3; p < 0.001) ^15^. Similar interventions had been previously conducted and discussed regarding their feasibility, applicability, and increased participant confidence in using car seats ^47^.

Virtual reality (VR) has also proven to be an important strategy for reducing traffic accidents, particularly discussed in other studies as beneficial for reducing pedestrian accidents among children ^48^ ^,^ ^49^. Although the study using VR showed weaker evidence compared to other studies in our review, the results described a reduced probability of accidents post-training and increased likelihood of checking traffic in both directions before crossing the first traffic lane ^31^. This underscores the need for further trials validating the efficacy of VR.

Initiatives aimed at educating children about falling events, whether to prevent or reduce harm, are also important, as these events hold a high prevalence in childhood and cause pediatric unintentional emergencies and urgencies, representing a large part of hospitalizations, along with burns, drownings, and traffic accidents ^50^. Among the most common accidents in childhood, falls were identified as the leading cause of hospitalization in all age groups from 0 to 15 years ^51^. Given the above, interventions such as the Safe Fall program for promoting actions involving physical education classes, with the practice of games and exercises for the assimilation of safe positions, have achieved results that reduced the risk of injury and increased the adoption of safe positions in a fall event ^29^ ^,^ ^30^. In children aged 6 to 12 years, the risk of injury, initially estimated at over 90%, was reduced to levels ranging from 8.7% to 18.3% due to the students’ natural responses to falls ^30^. Similar strategies to the Safe Fall program, which focus on adopting proper body positions, have shown promising results in reducing fall events and their severity ^52^ ^,^ ^53^.

Therefore, the implementation of strategies for preventing childhood accidents is essential in promoting the health and well-being of this population, considering that external causes are among the leading causes of death in pediatrics. The applicability of some studies are limited to specific contexts and conditions of their samples, requiring critical reflection for the development of similar models, given the existence of cultural, socioeconomic, and geographical heterogeneity, for example.

### Limitations

As this is a literature review, the limitations of the included studies may influence the outcomes of our research. The absence of control groups for result comparison is noted ^11^ ^,^ ^28^ ^,^ ^31^ ^,^ ^33^ ^,^ ^38^, as well as the lack of sample groups standardization, size, and rigor during the conduction of some studies ^16^ ^,^ ^18^ ^,^ ^20^ ^,^ ^33^ ^,^ ^36^ ^,^ ^48^ ^,^ ^54^. Some studies relied on self-reported data collection from individuals, which may underestimate or overestimate the real results ^12^ ^,^ ^14^ ^,^ ^21^ ^,^ ^22^ ^,^ ^27^ ^,^ ^31^ ^,^ ^48^ ^,^ ^54^. The lack of a standardized, validated, or fully understood instrument by the research team is also a limiting factor ^11^ ^,^ ^16^ ^,^ ^17^ ^,^ ^18^ ^,^ ^24^ ^,^ ^25^ ^,^ ^28^ ^,^ ^32^ ^,^ ^33^ ^,^ ^35^ ^,^ ^39^ ^,^ ^48^ ^,^ ^54^. Other results have limitations that hinder generalization and replication in other populations due to their strong local cultural factors and socioeconomic bias ^14^ ^,^ ^15^ ^,^ ^16^ ^,^ ^19^ ^,^ ^37^ ^,^ ^39^. Some studies were conducted during the COVID-19 pandemic, limiting participant structure and follow-up ^13^ ^,^ ^36^ ^,^ ^40^.

## Conclusion

This systematic review synthesizes innovative and interactive educational strategies that have demonstrated effectiveness in preventing childhood accidents within the community context. Among the identified effective strategies are playful approaches aimed at children, such as the use of stories, games, and educational activities that respect the cognitive abilities of each age group. For caregivers, educational actions using visual and interactive methods stand out, including lectures, quizzes, and group activities focused on disseminating technical knowledge in an accessible and efficient manner.

The implementation and maintenance of these strategies face significant challenges, including long-term evaluation, parental educational attainment, socioeconomic vulnerabilities, and diverse geographic and cultural contexts. These factors influence the effectiveness and sustainability of preventive programs. Therefore, future studies should use validated instruments and rigorous criteria for participant inclusion and exclusion, as well as conduct prolonged follow-ups to assess the ongoing efficacy of interventions.

The findings of this research provide a basis for planning health education programs aimed at preventing unintentional injuries in childhood, benefiting both children and their caregivers within the community context.
